# VUB-CYBERLEGs CYBATHLON 2016 Beta-Prosthesis: case study in control of an active two degree of freedom transfemoral prosthesis

**DOI:** 10.1186/s12984-017-0342-y

**Published:** 2018-01-03

**Authors:** Louis L. Flynn, Joost Geeroms, Tom van der Hoeven, Bram Vanderborght, Dirk Lefeber

**Affiliations:** Robotics and Multibody Mechanics, Flanders Make, Vrije Universiteit Brussel, Pleinlaan 2, Brussels, B-1050 Belgium

**Keywords:** Prosthesis, Transfemoral, Knee, Ankle, Active, CYBATHLON

## Abstract

**Background:**

Here we present how the CYBERLEGs Beta-Prosthesis was modified with a new control system to participate in the Powered Leg Prosthesis event, and to report on our experience at the CYBATHLON 2016 which was held in Zurich, Switzerland in October 2016. The prosthesis has two active degrees of freedom which assist the user with extra joint power at the knee and ankle to complete tasks. The CYBATHLON is a championship for people with disabilities competing in six disciplines, using advanced assistive devices. Tasks for CYBATHLON 2016 were chosen to reflect everyday normal task such as sitting and standing from a chair, obstacle avoidance, stepping stones, slope walking and descent, and stair climbing and descent.

**Methods:**

The control schemata were presented along with the description of each of the six tasks. The participant of the competition, the pilot, ran through each of the trials under lab conditions and representative behaviors were recorded.

**Results:**

The VUB CYBERLEGs prosthesis was able to accomplish, to some degree, five of the six tasks and here the torque and angle behaviors of the device while accomplishing these tasks are presented. The relatively simple control methods were able to provide assistive torque during many of the events, particularly sit to stand and stair climbing. For example, the prosthesis was able to consistently provide over 30 Nm in arresting knee torque in the sitting task, and over 20 Nm while standing. Peak torque of the device was not sufficient for unassisted stair climbing, but was able to provide around 60 Nm of assistance in both ascent and descent. Use of the passive behaviors of the device were shown to be able to trigger state machine events reliably for certain tasks.

**Conclusions:**

Although the performance of the CYBERLEGs prosthesis during CYBATHLON 2016 did not compare to the other top of the market designs with regards to speed, the device performed all of the tasks that were deemed possible by the start of the competition. Moreover, the Pilot was able to accomplish tasks in ways the Pilot’s personal microcontrolled prosthesis could not, with limited powered prosthesis training. Future studies will focus on decreasing weight, increasing reliability, incorporating better control, and increasing the velocity of the device. This is only a case study and actual benefits to clinical outcomes are not yet understood and need to be further investigated. This competition was a unique experience to illuminate problems that future versions of the device will be able to solve.

## Background

The CYBERLEGs Beta-Prosthesis is a transfemoral prosthesis with two active degrees of freedom, one in the knee and one in the ankle, designed primarily to help those with limited ambulation ability using standard prostheses due to weakness from advanced age or complicating illness. The prosthesis was originally created as a part of the larger CYBERLEGs Project, which combines this prosthesis system to replace a lost limb in parallel with an exoskeleton to assist the sound leg and hips, and a sensory array to control both systems. The end goal of the complete CYBERLEGs system was to assist those who have both a loss of a limb and weakness in the remaining limb to regain walking function and improve walking behavior. Here we have taken the CYBERLEGs prosthesis out of the complete CYBERLEGs environment and adapted it to function independently, including an entirely new control system, for use in the CYBATHLON 2016 competition held in Zurich, Switzerland in October 2016 [1].

Although the device has two powered joints, it is designed to allow a high level of passive behavior during the gait cycle through the use of passive components, either built into series elastic actuators, or springs that are inserted and removed from interaction by locking mechanisms. Through the use of these passive energy storage components, it is possible to, with simple control, create energy efficient gait cycles for normal walking [2, 3]. Moreover, the prosthesis is capable of providing the full ankle and knee torques during walking, as well as a large percentage of the torque required for normal sit to stand and stair climbing activities [4].

The CYBERLEGs Beta-Prosthesis was originally controlled using a gait intention detection system [5], which incorporated an array of IMU’s and pressure insoles for accurate center of pressure measurements of both of the feet. A system comprised of so many sensors and requiring many processing techniques was deemed too complicated for the competition and was replaced by a new, simpler control system which is described.

The CYBATHLON 2016 competition was designed to test the ability of everyday activities that anyone might face during the day, such as sitting and rising from a chair, maneuvering through obstacles, walking up and down steep slopes, and stair climbing and descent. By comparing performance in a parallel track obstacle course race, the competition was designed to gauge state-of-the-art systems in accomplishing these tasks [1]. The competing teams used a variety of currently available active (Power Knee, Ossur), microcontroller (Rheo Knee XC, Össur and Genium X3, Otto Bock), and passive (Total Knee, Össur) devices and the competition also showcased a few new devices, such as the Rise Legs (Rise), AMP-Foot 4 (VUB) [6], Xiborg, and Ortokosmos (Metiz Hyperknee) offerings.

This paper presents first a brief overview of the workings of the CYBERLEGs Beta-Prosthesis as well as some key aspects of the design that were adapted specifically for the tasks of the Powered Leg Prosthesis event of CYBATHLON 2016. The control and representative behavior of the prosthesis during each of the tasks of the CYBATHLON is then presented. A discussion about the particular design choices and results from the CYBATHLON controller, including a discussion of implications for future developments, follows.

## Methods

The CYBERLEGs Beta-Prosthesis is not built like a standard passive prosthesis in use by most people today, but includes motors in both the knee and the ankle for active energy input to the joint. It utilizes a unique combination of series elastic motors and also exploits locking spring mechanisms to achieve energy efficient regular walking with enough capability to perform other tasks. A short description of the joint construction is followed by the electronics system which was completely redone for the CYBATHLON. The Pilot is an integral part of the system, introduced after the electronics, followed by the state machine based control system and how it was run for each task.

### The CYBERLEGs Beta-Prosthesis

**Fig. 1:**
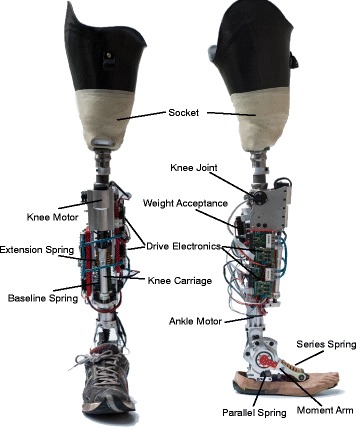
The Beta Prosthesis. The Beta Prosthesis as used during the CYBATHLON without the protective covers. Important components of the prostheses are labeled

The CYBERLEGs Beta-Prosthesis is an integrated transfemoral prosthesis containing independent active drives in both the knee and the ankle. These active drives allow the joint to provide both positive and negative work during a motion. Both the knee and the ankle are designed with series elastic actuators, allowing dynamic forces from the device to have a larger influence over its behavior. In this version, spring stiffnesses for both the knee and the ankle were chosen based on the torque angle characteristics of a 80 kg person walking at the ’normal’ velocity of 4.8 km/h, as defined by Winter. [7] The prosthesis weighs around 6.5 kg, including the socket, shoe, electronics, and cover, which is considerably more than most prostheses, especially considering the batteries are external, but the device itself has about the same weight and inertial distribution as a normal leg. An image showing the device can be found in Fig. 1, with the major components labeled.

#### Ankle design

**Fig. 2:**
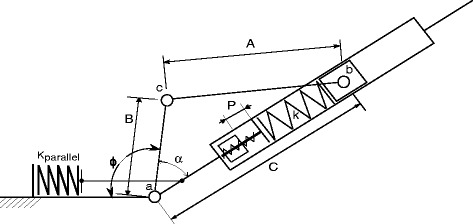
MACCEPA Ankle Schematic. Schematic of a MACCEPA using rigid linkages. The main motor drives the moment arm (b) around the ankle joint (a). The moment arm is displaced by an angle *α* with respect to the spring axis, which is defined as the neutral axis of the actuator. This displacement compresses the main MACCEPA spring (k) along the foot shaft (C), creating an ankle torque. The parallel spring (*K*_*parallel*_) is engaged during dorsiflexion, but is not in contact during plantarflexion. The pretension of the ankle (P) was constant throughout the competition. Note that *α* is a combination of the output angle and the moment arm angle *ϕ*, which is not influenced by the ankle output

The ankle is a design based on a MACCEPA actuator with a parallel spring system. The actuator of this device has been previously discussed in [8, 9]. The additional parallel spring was added to this system to provide stability when unpowered as well as reduce the peak torque required by the ankle actuator which allowed for a reduction of the gear ratio of the actuator and increased velocities. A schematic of the ankle actuator can be found in Fig. 2.

In this ankle, the main motor is housed within the shank of the device. This motor is attached to a 33:1 planetary gearbox which is in turn driving a 10:1 hypoid drive gear. The shank can be slid relative to the knee to adjust for height as well as rotated for ankle and knee joint parallelism. This motor drives a moment arm which drives a crank slider to compress the series spring. This creates the joint torque of the device. The parallel spring is unilateral and engages at approximately 3 degrees of dorsiflexion. Key component values are found in Table 1.

**Table 1:** Selected prosthesis characteristics used during CYBATHLON 2016

Property	Value	Units
System voltage	24	VDC
Ankle motor	Maxon EC-4Pole 30	200W
Ankle actuator	130	N/mm
Spring constant (*k*)		
Ankle parallel spring constant	94.20	N/mm
Ankle max torque	130 (*@*15A)	Nm
Ankle continuous torque	30.6	Nm
(no parallel spring)		
Ankle total gear ratio	330:1	
Knee motor	Maxon EC-i 40	50W
Knee max torque	∼ 70	Nm
Knee continuous torque	∼ 55	Nm
Knee gear (Planetary) ratio	5.8:1	
Knee ball screw lead	2	mm/turn
Knee range of motion	0 to 95	deg
Baseline spring constant (*K*_*BL*_)	10.7	N/mm (each spring)
Baseline spring mass	19.7	g
Extension spring constant (*K*_*EX*_)	89.1	N/mm (each spring)
Extension spring mass	15.6	g
Weight acceptance	300	N/mm
Spring constant		
Weight acceptance	90	g
Spring weight		
Prosthesis overall mass	∼ 5	kg

#### Knee design

The knee of the system is composed of two major components, the Knee Actuator (KA) and the Weight Acceptance (WA). The WA is a stiff spring that is driven by a non-backdrivable screw feed so it can be positioned to either interact or avoid contact with the knee joint. The non-backdrivability allows it to create large extension torques without requiring power. This device is used for stiff knee behaviors, such as the weight acceptance phase of the gait cycle or when a straight and stiff leg is desired. The WA can be seen on the back side of the prosthesis in Fig. 1.

**Fig. 3:**
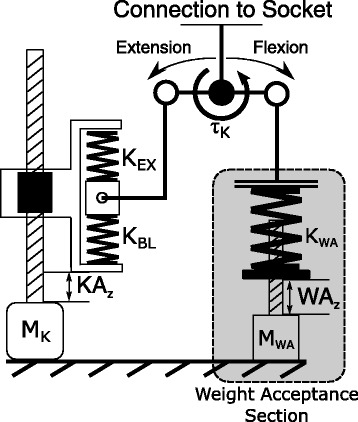
Beta-Prosthesis Knee Schematic. Schematic of the knee system showing the knee drive and carriage and the Weight Acceptance side. The connection to the carriage allows both flexion and extension torques to be created by adjusting the position of the carriage (*KA*_*z*_) while the WA side is unilaterally constrained allowing only extension torques to be created. The knee angle at which the WA is engaged is changed by varying *WA*_*z*_

The KA provides the main flexion and extension torques for the majority of the gait cycle. This is done through a series elastic actuator actuating on a push/pull rod that flexes the knee joint. This actuator has two different spring constants which provide different stiffness behaviors between flexion and extension torques. This type of architecture has been shown in simulation and on the test bench to have a lower energy consumption than a stiff system due to the capability of storing and releasing energy in the series spring of both the WA and the KA systems [2]. A schematic of this device can be found in Fig. 3. In this Figure, it can be seen that changing the position of the carriage (*KA*_*z*_) can create an extension or flexion torque, but the WA position (*WA*_*z*_) can only provide an extension torque due to the unilateral constraint at the WA spring.

### Prosthesis attitude detection

The prosthesis was controlled by a finite state machine, which was driven by inputs from the prosthesis and from the thigh of the pilot. The majority of the state changes required for the controller were determined by inertial rate gyros found on the pilot’s thigh. This device was used to detect a number of behaviors, for example an intentional hip eversion to initiate stair climbing. This signal was analyzed using a Phase Plane Invariant method of the type of [10] to determine the position of the hip while reducing error due to gyro drift. For many of the states, the prosthesis kinematic values could be used to determine state transitions, such as knee angle or ankle angles. The ankle MACCEPA actuator was also used to estimate ankle torque from foot placement, which was used as a trigger for some of the states. The exact use of how these signals are used to trigger state transitions can be found in “Events and control methods for the CYBATHLON” section.

Note that the prosthesis starts and can at any time be commanded, either through an error detection or deliberate intention, into the idle state. The idle state is the extended locked position with the WA raised and the knee carriage at full extension, which is considered to be the safest, most stable, and most predictable prosthesis state.

### Prosthesis electronics

**Fig. 4:**
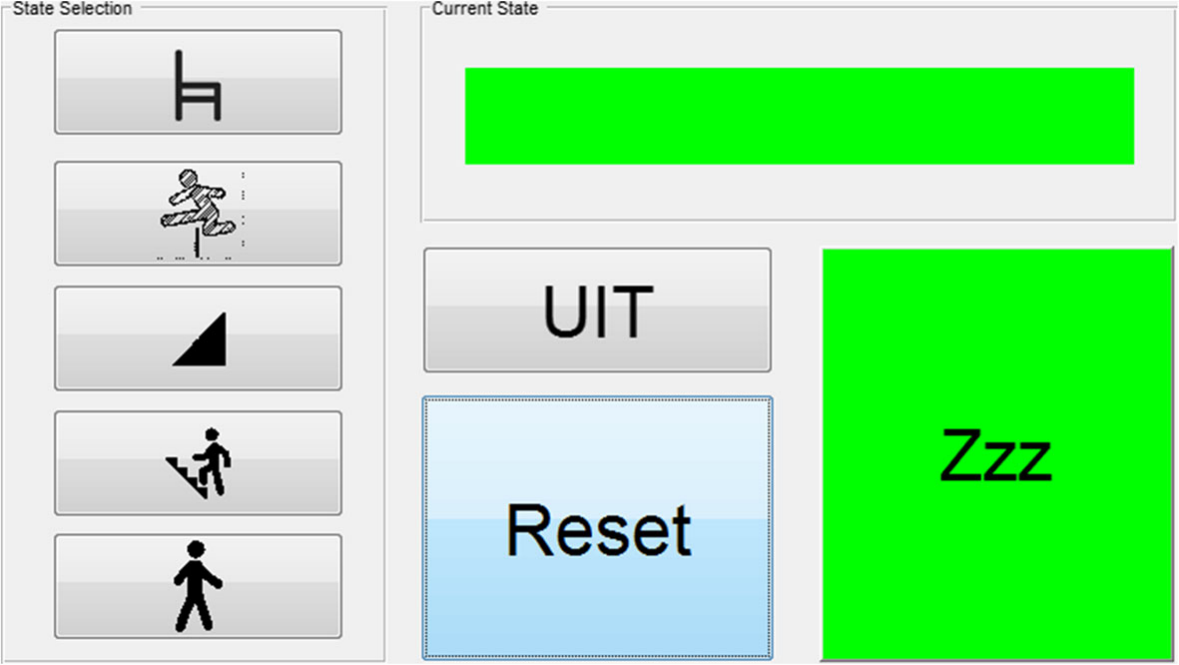
VUB CYBERLEGs Pilot GUI. Image showing the GUI screen as seen by the pilot. The screen was worn on the wrist (see Fig. 5), and allows the pilot to control the state of the prosthesis or to reset the device. The top green section turns red in an error state and displays the error message. The green ’Zzz’ button can be pressed at any time to send the device to the Idle state. The button ’UIT’ (Off in Dutch) is the motor disable button, used when the prosthesis needs to simply be dormant. The five prosthesis functions are seen of the left (from top to bottom), corresponding to the sit-to-stand, hurdles, slope walking, stair climbing, and normal walking states needed for the CYBATHLON

**Fig. 5:**
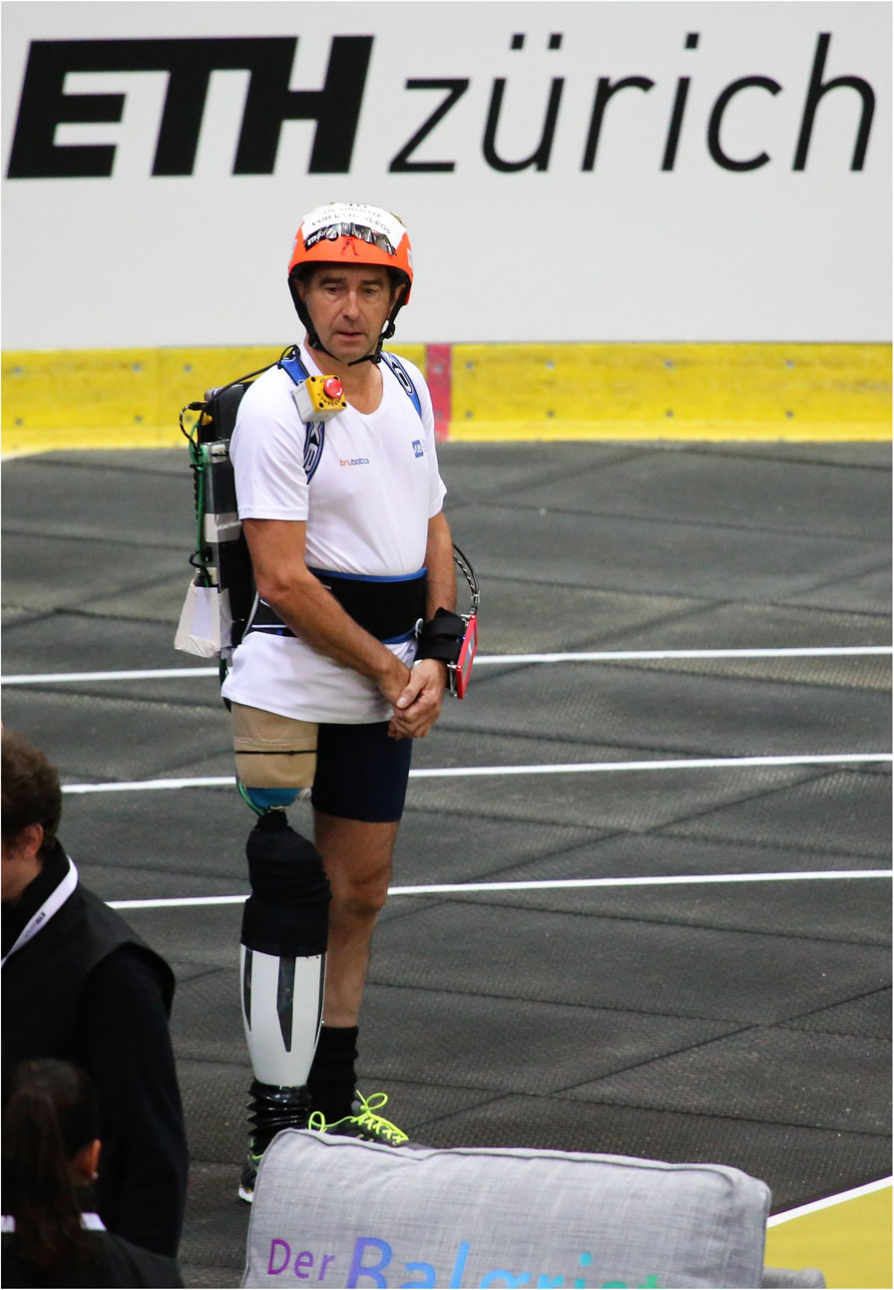
The VUB-CYBERLEGs pilot. Our pilot, Michel de Groote wearing a complete VUB-CYBERLEGs Beta Prosthesis system during the CYBATHLON. The system consists of the prosthesis, a backpack with the computer and battery, and an arm mounted touchscreen control

The prosthesis utilizes four custom made EtherCat slaves [11] which are capable of reading all of the sensors of the system including SPI, digital I/O, and analog I/O interfaces. Three of the boards are also populated with an ESCON 50/5 Module (Maxon Motor ag, Sachseln, Switzerland) for motor driving. The fourth board was used for additional sensor input and provided a backup system that could replace one of the other driver boards if necessary. The EtherCat master was a laptop computer running Simulink (Mathworks, Natick MA, USA) and TwinCat software (Beckhoff Automation, Verl, Germany) to create a real-time EtherCat master on standard PC hardware. The EtherCat control loop was run at 1000 Hz, reading the entire prosthesis state and creating command velocity commands for the motor drivers. The low level motor drivers were configured in a closed loop velocity mode sampling at 5.36 kHz, tracking the velocity signal created by the main controller. Incremental encoders were located on each motor and joint outputs were measured by 14 bit magnetic absolute encoders. Angular velocity of the hip was measured by two analog output 1500 deg/sec 2DOF rate gyros oriented with a common axis along the longitudinal axis of the leg. The laptop was worn in the backpack of the system when running autonomously, and would be run from the bench while running tethered experiments. The prosthesis high level control was directed by a wrist worn touchscreen system which allowed the pilot to select the high level action he wished to use or perform actions such as reinitializing or disabling the prosthesis. This touchscreen diagram can be found in Fig. 4 and an image of how the touchscreen was worn can be found in Fig. 5.

The prosthesis was run with a 24V battery housed in the backpack, which is half of the original design voltage. This was done to reduce battery size and leave overhead for the motor drivers to protect from over voltage conditions during regenerative periods such as slope and stair descent. This limited the maximum velocity of the device to approximately half of the original design velocity. An emergency stop was placed on the strap of the backpack and a current limiting breaker was placed on the backpack for the competition, both of which would immediately cut all power to the system.

### The pilot

The subject of the tests, who in the parlance of the CYBATHLON is named the pilot, was 58 year old Michel De Groote seen in Fig. 5, a transfemoral amputee since having osteosarcoma treatment in 1989. Michel weighs 60 kg without his prosthesis and stands 1.70 m tall. His current prosthetic limb is an Otto Bock 3C98-3 C-Leg paired with a standard passive ESR ankle. The pilot was recruited by our sponsor, VIGO International (Wetteren, Belgium), who also provided the socket system and prosthesis alignment for CYBATHLON 2016.

Michel has a relatively high femoral amputation limiting his ability to balance or apply large hip torques. This makes it extremely difficult to take stairs step over step or to balance on one leg with his current prosthesis, but in terms of the goals of CYBERLEGs this makes him an interesting test candidate. He was able to come to the lab and use the prosthesis around 14 h total, split across 5 different sessions of training and tuning. This amount of training is relatively short especially considering the amount of trust the pilot must have in the prosthesis to make it function correctly and the large weight and difference in functionality from his standard prosthesis.

### Events and control methods for the CYBATHLON

The CYBATHLON 2016 Leg Prosthesis Race allowed pilots to compete on parallel tracks to complete several tasks related to daily life. These six different tasks consisted of the Sit-to-Stand (StS), hurdle navigation, slope climbing and descent, stepping stones, tilted path, and stair climbing and descent. Pilots were allowed 4 min to complete the entire parkour. Here we discuss the behavior and control of the prosthesis while performing each of these tasks.

At the beginning of each task the pilot selected an appropriate state machine to use for the task using the touchscreen. This allowed us to change the behavior of the prosthesis without having to develop a new gait intention detection system, and give the pilot a concrete indication about about which state machine was in operation. Each of these state machines consisted of trajectory generators for the KD, ankle actuator, and WA systems. These trajectories were either a torque or position trajectory, depending on the type of controller the state machine desired. The generator used a piecewise linear calculator that, upon entry of a new state, used the current position of the device to create the new trajectories and avoid discontinuities in the desired motor position. The torque or position rise rate, fall rate, and amplitude, were determined by experiment or estimation from modeling. Estimations of the positions of the actuators were initially calculated by looking at human data and dividing the task into states where the behavior of the system did not drastically change, the threshold for each of the states was then determined experimentally after initial guesses were made.

While the prosthesis was in position control mode, the motor position *KD*_*z*_, the ankle moment arm position (*ϕ*), or *WA*_*z*_, rather than the output kinematics or output torque of the system, was controlled with closed loop feedback. This method tracks a predetermined SEA rest position allowing the passive spring and device geometry to determine the overall joint impedance. This is different from the techniques of many powered prostheses which rely on output trajectory tracking with a true impedance controller [12, 13], instead relying on the natural impedance of the system to dominate.

The use of torque control mode was determined to be necessary during some tasks when position control mode failed to produce satisfactory results. Sit to stand was the first task where it was determined that being able to change the velocity of sitting to stand and stand to sit would be beneficial, which the position control system would not allow.

The following sections describe each of these state machines for each of the events, including the type of controller used for each state as well as the required conditions for state transitions.

#### Sit to stand

The pilot must sit and stand from a standardized chair, fully removing the feet from the ground when sitting. After each standing attempt, the pilot must then take a step ahead 1.20 m to a line and step back to the chair before sitting again. Use of hands is allowed to rise from the seat, but the seat back should not be used.

**Fig. 6:**
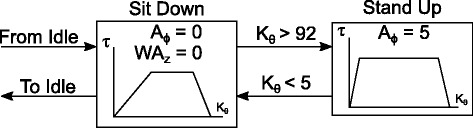
State chart of the Sit to Stand mode. Sit to stand used two torque trajectory based states, providing assistance with different torque profiles depending on whether sitting or standing. The ankle motor moved so the resting angle would allow for a flat foot while sitting

Figure 6 shows the sit-to-stand mode of the state machine, showing that it contained two different torque profiles based on whether the pilot was standing or sitting. Both of these states provide an extension torque, assisting during Sit to Stand and braking during Stand to Sit. The WA was not used during this function, and so was set to its lowest position. The ankle was moved using the position control to a slightly plantarflexed position, meaning the ankle moment arm angle (*ϕ* in Fig. 2) is set to -5 degrees with respect to the neutral position, so that the foot would lie flat on the ground while sitting and returned to straight while standing. The states were switched based on the knee angle.

#### Hurdle navigation

This section consisted of four hurdles, the first and last consisting of a horizontal bar at 200 mm from the floor, and a second bar at 1500 mm from the floor. The middle two hurdles consisted of a single horizontal bar at 350 mm from the floor. The width of the hurdles was 900 mm and spaced apart at intervals of 600 mm. The pilot was required to pass through the obstacles without knocking down any of the horizontal bars and without using their hands.

**Fig. 7:**
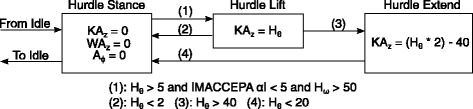
State chart of the Hurdle navigation system. The hurdles were controlled through the angle of the hip (*H*_*θ*_) with respect to the standing position. Initialization of the hurdle lifting begins with a hip velocity (*H*_*ω*_) over a certain threshold. Lifting and extending phases were done at different rates, the knee angle providing the trigger the switch between states.

Hurdle navigation consisted of bending the prosthesis knee when the hip was bent so the prosthesis would clear the hurdle. This action was triggered by a threshold on the velocity of the hip flexion (*H*_*ω*_) which then would then command the knee to bend by relating the hip angle (*H*_*θ*_) to a position of the KA carriage. The relationship between the hip angle and carriage position was different for the lift and extension states. A full schematic of the hurdle navigation, including thresholds and command positions can be found in Fig. 7.

#### Ramp climbing and descent

The ramp climbing and descent section included climbing a steep 20° incline, opening and closing a door on the platform, then descending a 15° slope without the use of handrails.

**Fig. 8:**
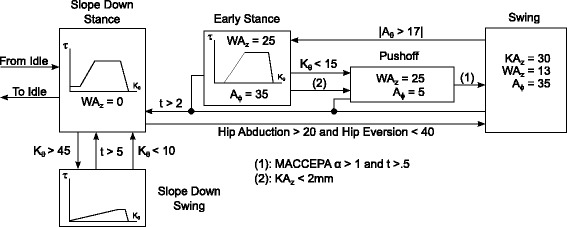
State chart of the Ramp Climbing and Descent system. From the slope down state it is possible to descend slopes or enter into the ascent phases with an abduction of the hip

Entering the slope climbing state machine from the idle state, the prosthesis was set in the slope descent mode. By descending a slope and allowing the knee to flex to a certain angle, the slope decent extension phase would begin and apply a different torque profile to the knee joint. During the slope descent the ankle angle was set to neutral, but was able to adapt to the slope due to the passive compliance of the system. To trigger the slope ascent, the pilot would perform a hip abduction movement which would place the leg into the slope swing phase. The slope swing phase is a position controlled state where the positions of *KA*_*z*_, *WA*_*z*_, and *A*_*ϕ*_ are predetermined. To trigger the stance state of the slope climbing, the ankle angle must be deflected beyond a set angle. Because the motor position is constant, this corresponds to a known ankle torque, ensuring the ankle is on the surface and weight is transferred to the prosthesis. At this moment the KA applies a torque profile to the knee to assist with climbing the slope and reaching full leg extension. The WA is also raised to allow the pilot to push on it during pushoff and the ankle remains highly dorsiflexed. The pushoff phase is reached at a determined knee extension, where the ankle is then plantarflexed to provide pushoff. Note that if the device remains in any of the stair ascent states for longer than a timeout period (*t*), the device returns to the slope down state. A full schematic of the ramp climbing and descent control, including thresholds and command positions can be found in Fig. 8.

#### Stepping stones

The stepping stones task was a path of seven half cylinders placed with 600 mm intervals in the direction of walking and 750 mm in lateral movements. Only one foot could touch a stone, and the pilot was not allowed to touch the ground between the stones or any other hand rails.

Because the stepping stone task was not possible to safely maneuver for our pilot, due to the aformentioned balance problems due to a short residual limb and lack of balance specific adaptations like ankle inversion/eversion, we did not attempt this in the competition and therefore did not have a control section in the state machine.

#### Tilted path

The tilted path was a series of two platforms with a leading and trailing edge sloped at 18° and a width of 2000 mm. The center of the platform was sloped from the floor on one side to 300 mm height at the other side. The center slopes were alternated first sloping down toward the right and then toward the left. The two platforms were separated by 300 mm.

The tilted path could be handled by the pilot through normal walking, or if he desired it could be navigated with a leg that was in the idle state and therefore there was no tilted path specific state machine.

#### Stair climbing and descent

The stair climbing task required the pilot to climb and then descend a set of 6 standardized stairs without use of a handrail. Only one foot was allowed on each stair. Upon the first completion of an ascent and descent, the pilot was to pick up two plates with item on them from a table, and return over the stair case and place the plates on another table and finally return over the staircase one final time.

**Fig. 9:**
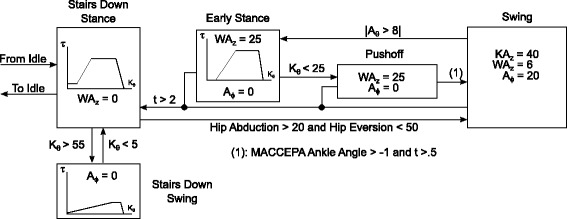
State chart of the Stair Climbing system. The technique of this state chart is similar to the one of slope climbing (Fig. 8)

The state machine for stair climbing, which can be found in Fig. 9, was similar to the one for the slope climbing (See Fig. 7), mainly because the angle of the slope section was so large it essentially was much like climbing stairs with a different ankle angle. The ankle angle was held neutral for stance and pushoff, while during swing it was changed to a 20 degree dorsiflexion. All other commands were essentially the same between the two systems. Here again the compliance of the ankle was used in determining proper weight transfer to the new stance leg. Once again the ankle was used as a torque sensing device to detect foot fall and weight transfer on the new stance stair and for foot liftoff.

## Results

The tasks that were attempted at the CYBATHLON were performed in the lab of the Vrije Universiteit Brussel, in Brussels, Belgium and the behavior of the prosthesis was recorded. The computer was not recording data during the actual competition to reduce the small possibility of errors occurring due to the saving functions and to simply reduce the load on the computer to ensure it was running at peak performance. The tests were designed to best emulate the behavior during the actual competition. These tests were all with the permission of the VUB Medical Ethics Commission (B.U.N. 143201526629). All data from the prosthesis was collected at 100 Hz and analyzed in MATLAB. The current values were then filtered using a low-pass, zero phase shift, two pole Butterworth with a cutoff frequency of 10 Hz.

The knee torque was determined using two different methods. The first was through an inverse kinematics model of the knee which is possible because the knee actuator is a series elastic device and by measuring the drive side and output link positions, the torque of the joint can be determined within the linear region of the series elastic spring. Outside of this region it is possible to estimate the torque of the actuator using the current of the motor to determine the output torque. In this method the current of the motor is used to determine the force applied by the ballscrew on the actuator, which is directly related to the output knee torque by the kinematics of the knee. These two methods show good consistency when the motor is being driven, but when being backdriven the current does not correspond to the output torque due to unmodeled efficiency losses during backdriving and driver reverse current capability, and so there are large deviations in the two methods [14]. It should also be noted that here when the knee carriage is at its lowest position, there is a slight extension torque on the knee joint. This is just to add a bit of stiffness in the fully extended position if the WA is not in place.

### Sit to stand

**Fig. 10:**
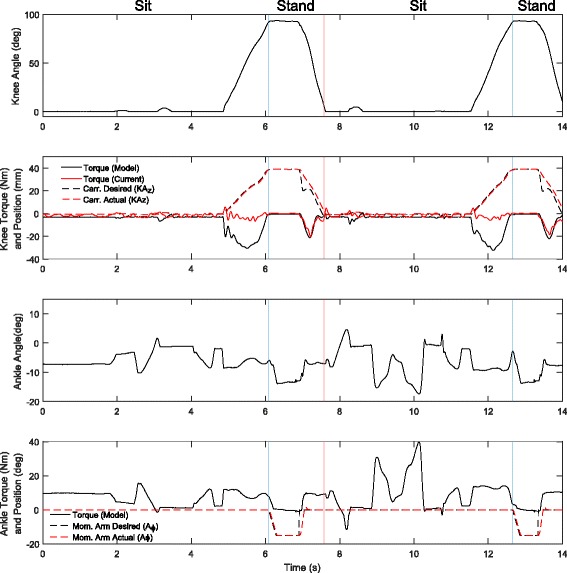
Sit to Stand Torque/Angle Characteristics. Knee and ankle angle, torque, knee carriage (carr.), and ankle moment arm position during sit-to-stand and stand-to-sit experiments. The sitting phase starts when the knee flexion hits 5 degrees. The knee is flexed to its maximum of 95 degrees and desired torque is brought to zero. The knee extension assistance is then started when the knee is extended past 93 degrees. Trace represents one sit to stand to sit cycle

The pilot followed the sit-to-stand procedure and the knee angles and knee torque are presented in Fig. 10. The knee flexion is defined as a positive angular displacement, and therefore extension torques are defined as negative. Large negative torque can be seen during the sitting phase in the kinematic displacement model, but because this motion backdrives the knee motor, the actual motor current is very low and the current model does not show the correct output torque. While standing the prosthesis gives a modest 20 Nm assistive torque, and because this is a net positive work action, the current model agrees with the kinematic model.

The ankle moment arm is placed with a slight plantarflexion while in the sitting position. This allows the foot to sit flat on the ground while in the chair. The larger peak torques seen at the ankle are due to the parallel spring during the step forward and step back that was required for the task.

Although not seen in this example, when the sit to stand action becomes too fast the torque assistance decreases due to the limited velocity of the knee motor. In this example the only time when the knee motor fails to track the desired position is at the beginning of the stand state, partially because of the reduced motor velocity due to a lower bus voltage, and also because the motor must move a long distance to produce the desired torque target due to the geometry of the highly bent knee. The lack of velocity of the actuators poses a particular problem in terms of the goal of accomplishing the the CYBATHLON in minimal time, but under normal use this velocity limitation is not such a large issue.

### Hurdle navigation

**Fig. 11:**
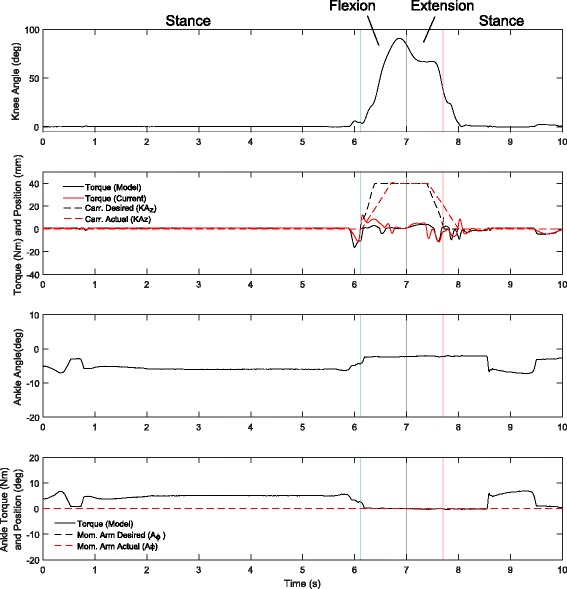
Hurdle Obstacle Torque/Angle Characteristics. Knee and ankle angle, torque, and knee carriage (carr., equivalent to *KA*_*z*_) and ankle moment arm position (Mom. Arm, equivalent to *A*_*ϕ*_) while navigating the obstacles. The knee torques remain low during the event, because the leg shank is kept relatively close to vertical as the hip is flexed. This flexion allows for easy navigation of the hurdles without resorting to manually flexing the limb with the hands. The ankle does not command different rest positions during the task, and only a small plantarflexion torque is seen during the stance phases

During the hurdle navigation the knee is flexed as a function of the hip flexion angle, allowing the pilot to control the knee flexion and extension by swinging his hip. Figure 11 shows the knee and ankle desired and actual behaviors during the test period. The hurdle navigation illustrates how the knee motor velocity is limited, showing a bit of tracking error in the desired and actual knee positions as he swings his hip quickly. Also a slight undulation of the knee occurs in areas of full flexion. This is due to the limited torque authority of the knee joint at high flexion due to the kinematics of the knee. At high flexion the knee Baseline Spring (*K*_*BL*_ in Fig. 3) stiffness dominates the behavior of the system and the motor must travel long distances to make changes in the torque of the knee. This coupled with the limited velocity of the knee motor means the knee is prone to vibrations at large flexion when it is not on the ground and the WA is not engaged. The ankle is held in the neutral position for the entire traverse, using only the passive behavior to provide ankle torque and compliance.

### Ramp ascent and descent

**Fig. 12:**
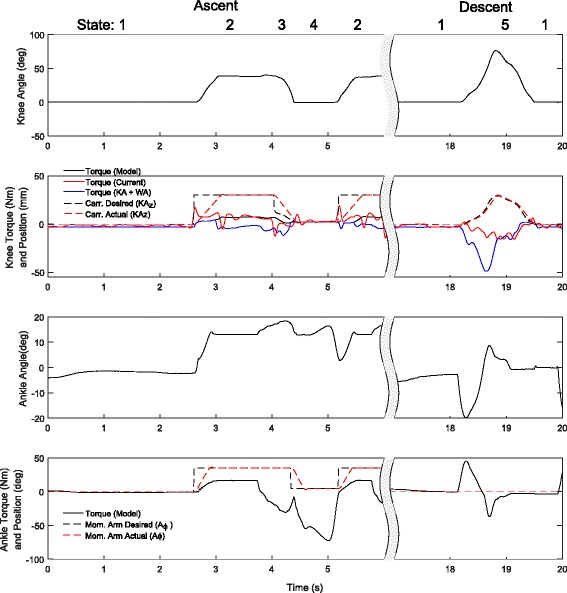
Slope Torque/Angle Characteristics. Ankle and knee angle, torque without WA (Black) and with WA (Blue), knee carriage (carr., equivalent to *KA*_*z*_), and ankle moment arm position (Mom. Arm, equivalent to *A*_*ϕ*_) for a representative slope climbing and descent cycle. The WA is used here to provide stiffness to the joint during ascent, although the amount the pilot used the WA for the task was lower than expected. The ankle dorsiflexes during the swing for foot clearance, and provides large torque during stance and pushoff. State 1 is the Slope Down Stance, State 2 is Swing, State 3 is Early Stance, State 4 is Pushoff, and State 5 is Slope Down Swing

Figure 12 shows the ascent of the slope taking four steps, and two steps down. Once again during the descent there is a large difference in the two methods of calculating the joint torque due to backdriving of the system. This is also a task where the WA system was utilized to provide a stiffer knee while flexed. The blue trace in Fig. 12 shows the torque due to the summation of the KD system and WA system. During the swing phase, the KA provides a flexion torque by actuating against the WA during this motion. The net result is an extension torque while the leg is loaded during the early stance phase, at a higher stiffness than would be otherwise.

The ankle is commanded to maximally dorsiflex against the parallel spring to provide large clearance of the foot during the swing phase. Then the ankle is set back to the neutral position during stance and pushoff. The result is decent clearance and the ability to provide high pushoff torque. The end rest position was determined by experiment.

### Stepping stones

The stepping stone task was not possible to safely maneuver for our pilot. This event requires that the pilot have excellent balance on the prosthetic limb, or have some sort of active control mechanism for accurate center of pressure. Because of the short residual limb of the pilot, he has limited balance control through the socket, and the prosthesis does not have inversion/eversion balance compensation to assist in this fashion. Adding active inversion and eversion of the ankle could potentially be very helpful for overall balance in this event.

### Tilted path

The tilted path could be handled by the pilot through normal walking, or if he desired it could be navigated with a leg that was in the idle state. Due to inconsistent initiation of the standard walking gait, the pilot chose to use the Idle state during the competition. Although stiff, using the Idle state to walk is possible through the passive compliance of the leg, as well as through the use of exaggerated hip motions. The passive flexibility of the ankle allowed the pilot to keep the foot flat against the surface in the fore/aft direction. The slope was not significant enough to require much evasive action. By approaching the task at an angle, the path could be as easily navigated as a flat floor. During the competition, some participants simply skipped over the obstacle with their device, only using the sound foot on the sloped surface and swinging the prosthesis over the entire obstacle. It is possible that this obstacle was not long enough or simply not steep enough to really provide a challenge to the pilots.

### Stair climbing and descent

**Fig. 13:**
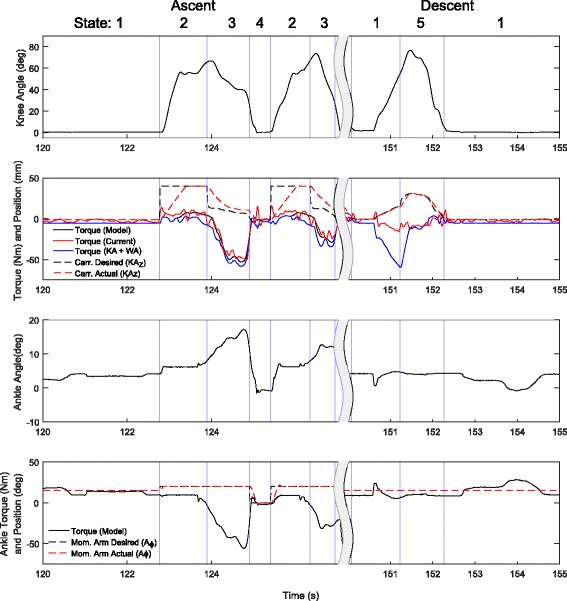
Stair Torque/Angle Characteristics. Ankle and knee angle, torque without WA (Black) and with WA (Blue), knee carriage (carr., equivalent to *KA*_*z*_), and ankle moment arm position (Mom. Arm, equivalent to *A*_*ϕ*_) for a representative stair climbing and descent cycle. During this test the handrail was used. State 1 is the Stair Down Stance, State 2 is Swing, State 3 is Early Stance, State 4 is Pushoff, and State 5 is Stair Down Swing

Our pilot could only perform this task using the handrail, and therefore only went once over the staircase once using the handrail, step over step. Figure 13 shows a cycle of six steps up and five steps down. Here the velocity limitation of the knee joint is apparent and it is limiting the torque output, except for the case of the first step which was taken slower and reached the maximum torque of the knee at that angle. The motor drivers of the knee were limited to 8A during this test, and the knee reaches this during the first step. The actual maximum extension torque for the device is about 60 Nm peak at about 30 degrees knee flexion.

Once again the WA is used during this task to provide some assistance with the bent knee. The result is only a modest 5 Nm extension at full flexion. Here it can be seen how the ankle was used to detect the transition from the Swing phase to the Early Stance. Also how the ankle is able to provide push off during stair ascent is clearly visible. Once again it is possible that better control techniques may be able to increase performance of this task [15], although implementation of controllers like these may run into limitations of the series elastic actuators [16].

## Discussion

CYBATHLON 2016 provided a perfect opportunity to improve the CYBERLEGs Beta-Prosthesis and gain a better understanding about what our device lacked with respect to real-world behavior by performing a standardized set of tasks. The competition also showed how a number of state-of-the-art devices compared with our device and with each other. It was apparent to us at the onset that our device was never intended to be run in a competition of such high intensity, and initial design decisions which were based on an entirely different target population would never allow the device to be highly competitive. Regardless, we determined that certain modifications could allow us to complete a number of the obstacles, and also allow us to gain insight to the benefits of powered prostheses in aggressive, active tasks.

Therefore the goal for competing in the CYBATHLON was never to win with this device, but rather to perform some of the tasks better than would be possible with a state-of-the-art passive device. Performing better not just in terms of task completion speed, but in terms of providing assistance to perform tasks more naturally and determining how to apply assistance to help perform these tasks for a regular user, and not necessarily a well trained athlete. In this goal there were definitely some things that were done well, and others that show limitations of the device and illuminate deficiencies that otherwise might have been missed.

Mechanically the prosthesis performed as designed and expected, without major failure. The controller, based on the combination of a limited set of sensors and user input, was able to fundamentally perform the tasks without a large amount of training. A necessary future addition to this device is an intention detection system as manually selecting state machines based on task is not ideal. Training time also has a large influence on the outcome of tests such as this. It is believed that if our pilot had much more time with a set control he would be able to optimize and utilize the device much more efficiently. In particular, we expect to see better use of the WA system during high extension torque operations. Regardless of these issues, we succeeded in creating a reliable state machine based system for control of the device which was able to perform most of the tasks of the CYBATHLON and have shown the active components of the device to be helpful in at least one aspect of each of the tasks.

It is very difficult to compare the behavior of of the CYBERLEGs Beta prosthesis to the other prostheses used in the competition because of a lack of data from those other devices doing the tasks from the competition. It would be interesting to really understand how other pilots were able to accomplish these tasks with empirical data, possibly using the CYBATHLON tasks as standard benchmarks for future studies. Another issue is that the level of fitness and familiarity of the device to the user has a large influence on the performance. When possible comparisons have been made to studies in the literature using these devices.

In the sit-to-stand task, the device performs quite well, providing a good amount of resistance while sitting and providing a solid assistance while rising from the chair. Only one other powered device, the Össur Power Knee, has been compared to current microcontroller based systems, [17, 18], but these papers show no benefit to the user in performing this task. These findings go against our experience with powered knee devices, where the patients who have used it find that any assistance at all in the prosthetic limb in the stand-to-sit and especially the sit-to-stand motion makes a noticeable difference in the ability to perform the action. It should be noted that in these papers the low level control of the prostheses, whether powered, microcontroller based, or passive were not able to be modified and may account for part of the difference in experience. The Wolf et al. [18] noted that the subjects who participated in the study were relatively healthy, young, and with no underlying complications, and it is possible that a different group, who may have a larger strength deficit for example, may gain more benefit from active assistance. In these papers there is no detailed analysis about what limitations the Power Knee might have in these studies from a control or technical point of view, rather focusing on clinical outcomes. Other devices have been tested with sit to stand properties [19], but no direct comparisons to how the joint torque related to behavior outcome were reported.

The current prostheses, with the exception of the Power Knee, cannot provide any positive torque while rising from the chair requiring the sound leg to provide all of the assistance. Michel has reported that when the assistive torque of the prototype is set correctly it feels as though he is being thrown out of the chair, greatly assisting the motion. Too much assistance can be a bit unsettling, but illustrates that the powered prosthesis really has an effect on at least the feel of rising from a chair. Also the foot is able to adapt to the ground level, allowing a more natural foot position while seated and while rising. Whether these benefits are seen as a reduction of work of the sound limb or greater body symmetry during the action remains to be determined.

During the hurdle navigation the prosthesis performed quite well, extending and contracting exactly as we wished. There are issues with the speed it is capable of performing flexion, and the weight of the device is another issue for all of the tasks where the prosthesis must be held high off the ground for extended periods. This was slightly mitigated through the use of a waist strap system, but during events of high hip flexion, it was necessary to hold the socket with the hands to ensure that it didn’t slip. The behavior of the knee was good for this task, compared to other devices in the competition where, to get the correct knee flexion, some pilots pulled on their knees with their hands. For a race such as the CYBATHLON this is a really good method to get through quickly, but as a general solution it is a bit of a clumsy action to have to perform, particularly if the user is not very strong in the sound limb.

During slope descent, there was a high sensitivity to torque rate due to the way the torque method was implemented. The balance between too much and too little initial torque and torque trajectory changed the behavior of the knee dramatically, although once a good setting was found the behavior was reliable, as long as the pilot could commit to the step. Hesitation at the beginning of the step would cause a reduction of knee torque and cause a stiff behavior. In descent cases such as this it may be better to model the knee as a damper and use techniques from current microcontroller devices [20] to handle this behavior. Indeed these types of dissipative actions are where microcontroller controlled damping systems excel.

Slope climbing also notably did not contain a large extension peak at the pushoff phase of climbing as stair climbing does, but this may be expected looking at biomechanical data (e.g. [21]) where there is an initial extension torque but then the knee torque changes into a flexion torque at the end of the stance phase. It is possible that with better control, possibly with a slope estimator [22], and training slope behavior could be greatly improved. The pilot did not use the WA system as much as was expected for this task. It was expected a high extension torque would be created by it at the beginning of the step ascent, using the spring to initiate leg extension by initiating a counter motion. This behavior may be simply because of a training issue, or simply not required for the task.

It was possible to perform step over step stair climbing and descent using a handrail and the torque curves in Fig. 13 show that the knee was able to provide a large assistive torque during climbing and dissipate a lot of work during descent. One issue is that he knee flexion at the beginning of stair ascent was not as large as it could be which may be caused by a combination of the prosthesis limitations and the pilot training. As it was set during the competition, the knee rests upon the WA when undergoing flexion during swing. This is so the pilot can load it during the beginning of the step up while the main actuator begins to gain torque. This was done this way because the main actuator cannot provide large torques at full flexion, and so it was hoped the WA could provide this during early step up. The pilot does not use this feature as much as we would have expected, and it is possible this can be changed with additional training. That said, the pilot cannot navigate stairs step over step at all with his every day prosthesis, and even though he had to relearn this task, the use of a powered prosthesis made it possible.

It should be noted that a well trained, strong individual can climb stairs step over step with all of the passive prostheses presented at the CYBATHLON. Pilots using most other devices (Genium, Orthokosmos, Rise, and three Ossur knees) completed this task without the use of handrails. Regardless, stair climbing is one function where having a powered knee is known to have a significant effect, reducing the required power generation of the sound limb, while performing slightly worse than the C-Leg in descent [20].

One omission from this summary is a discussion on level ground walking, which has be left out for a number of reasons. The first was that during the CYBATHLON, pilots were only required to take one or two steps between the different tasks; it was a very task oriented course and to switch to the walking state without an intention detection system would have meant manually switching state machines many times. Second the level ground walking methods are a bit more complex and are deserving of a more detailed analysis which, for brevity, is left out of this document.

## Conclusions

This case study is about the adaptation of an active prosthesis for use in CYBATHLON 2016, a competition held in October 2016 in Zurich, Switzerland. An existing prototype, the CYBERLEGs Beta-Prosthesis, was modified and new high and low level control systems and electronics were designed and built for the competition. Doing this allowed us to focus on making the prototype reliable enough to function for testing sessions and competition, as well as completing real-world tasks that displayed the functionality of the simplified controller and overall mechanics of the device. This competition served as a large motivation getting our device functioning well enough to complete the tasks and really allowed us to illuminate problems that future versions of the device will be able to solve.

While we were able to only officially complete four out of the six tasks, step over step stair climbing was possible with the assistance of a railing, which was a great improvement over previous implementations. In fact out of the five tasks we were able to complete, each had aspects that we feel characterize the increased capability of using a powered prosthesis. For example rising from a seat is a difficult task for someone who is weak, and we are able to experimentally measure an assistive torque that would not be there with passive devices. Assistance can be measured for stair climbing, and obstacle avoidance as well. The measurement of these assistive torques will allow a better understanding of how different torque profiles can help in performing tasks and normalizing gait. In addition, the use of compliant actuators allowed for automatic joint adaptation to sloped surfaces and also allowed for the use of the ankle as a torque estimation device for state triggers. All of these things are possible with the device, albeit at a low velocity. In the future we hope to bring these capabilities to a device that is able to compete with the current state-of-the-art in terms of speed and control through weight reduction and actuator redesign.

## Abbreviations

*τ*: Torque
A: Ankle
*A*_*α*_: Ankle moment arm angle with respect to the foot
*A*_*ϕ*_: Ankle Moment Arm Angle with respect the the shank, measured from the neutral position
*A*_*θ*_: Ankle angle
H: Hip
*H*_*ω*_: Hip angular velocity
*H*_*θ*_: Hip Angle
IMU: Inertial Measurement Unit
*K*_*θ*_: Knee Angle
KA: Knee Actuator
*K**A*_*z*_: Position of the knee carriage from the bottom of the ball screw
*t*: time in sec
WA: Weight Acceptance
*W**A*_*z*_: Position of the WA nut from the bottom of actuator
VUB: Vrije Universiteit Brussel
